# Trail Marking by Caterpillars of the Silverspot Butterfly *Dione Juno Huascuma*

**DOI:** 10.1673/031.011.5501

**Published:** 2011-04-25

**Authors:** Alfonso Pescador-Rubio, Sergio G. Stanford-Camargo, Luis E Páez-Gerardo, Alberto J. Ramírez-Reyes, René A. Ibarra-Jiménez, Terrence D. Fitzgerald

**Affiliations:** ^1^ Centro Universitario de Investigación y Desarrollo Agropecuario, Universidad de Colima, Tecomán 28100, Colima, México; ^2^ Facultad de Estudios Superiores Iztacala, UNAM, Laboratorio de Zoología, Tlanepantla 54090, Estado de México, México; ^3^ UNAM, Laboratorio de Zoología, Tlanepantla 54090, Estado de México, México; ^4^ Department of Biological Sciences, State University of New York at Cortland, Cortland, NY, 13045, USA

**Keywords:** foraging behavior, Nymphalidae, silk, social caterpillar, trail pheromone

## Abstract

A pheromone is implicated in the trail marking behavior of caterpillars of the nymphalid silverspot butterfly, *Dione juno huascuma* (Reakirt) (Lepidoptera: Heliconiinae) that feed gregariously on *Passiflora* (Malpighiales: Passifloraceae) vines in Mexico. Although they mark pathways leading from one feeding site to another with silk, this study shows that the silk was neither adequate nor necessary to elicit trail following behavior. Caterpillars marked trails with a long-lived pheromone that was deposited when they brushed the ventral surfaces of the tips of their abdomens along branch pathways. The caterpillars distinguished between pathways deposited by different numbers of siblings and between trails of different ages. Caterpillars also preferentially followed the trails of conspecifics over those of another nymphalid, *Nymphalis antiopa* L., the mourning cloak butterfly.

## Introduction

The Nymphalidae is the largest family of butterflies with over 6000 described species in 11 subfamilies (www.nymphalidae.net). The family contains a great diversity of species, many of which, such as the monarch, checkerspot, fritillary, and morpho, are among the most familiar of butterflies. The caterpillars of a number of species are social, with sibling aggregates often persisting for the duration of the caterpillar stage of the life cycle. One such species is the silverspot, *Dione juno huascuma* (Reakirt) (Lepidoptera: Heliconiinae), which is broadly distributed in Mexico, Central America, and South America. Five subspecies are recognized, with the subspecies *huascuma* occurring in Mexico (www.nymphalidae.net). Adult *D.j. huascuma* lays eggs in groups of up to 100 or more on the petioles and undersides of the leaves of vines in the genus *Passiflora* (Malpighiales: Passifloraceae) (Muyshondt et al. 1973). The caterpillars form tight aggregations at feeding sites on the plant, typically remaining together throughout the whole of their five caterpillar stadia, though they may form smaller subgroups as they mature (Muyshondt et al. 1973; Méndez-Martínez et al. 2007).

Alexander (1961) made detailed observations of *D. j. juno* in Trinidad, noting that its caterpillars were the most social of any of the 10 species of helioconiids that she examined. Although the caterpillars are nomadic, Alexander found that they move very little, resting adjacent to their feeding sites. Muyshondt et al. (1973) made similar observations of the feeding and resting behavior of *D. j. huascuma*, but, of particular interest, they also noted that when the caterpillars moved to new feeding sites they proceeded in a “solid column or line”. This behavior suggests they might employ a trail pheromone to hold the foraging column together. While general features of the foraging biology of the caterpillars of many species of the Nymphalidae have been documented, there have been no previous studies of the trail marking behavior of any of these species. The study reported here was undertaken to investigate the potential role that trail-based chemical communication might play in collective foraging behavior of the caterpillars of the silverspot.

## Materials and Methods

### Source of material and study location

Caterpillars of *D. j. huascuma* were collected in all caterpillar instars from natural populations occurring in the Estado de Mexico, Mexico. Laboratory studies were conducted at the UNAM Iztacala campus, Tlalnepantla, Mexico from January — April 2009.

### Response of caterpillars to pathways of conspecifics

Y-choice experiments were conducted to determine if caterpillars follow the pathways of conspecifics. Ten second instar caterpillars were allowed to move over a strip of vertically suspended paper approximately 30 cm long × 2 mm wide for one hour. The caterpillars were then removed and the strip cut into 1.5 cm long sections. One section was used to form one arm of a **Y** and another to form the stem. The other arm was made from a similar sized section that caterpillars had not walked on (Fitzgerald and Pescador-Rubio 2002). The response of 16 first and second instar caterpillars to the sections was observed by placing a caterpillar at the base of the stem of the maze and recording its choice of arm (Video 1). The position of arms was alternated each trial to preclude a positional bias. Maze sections and experimental caterpillars were used only once.

**Video 1.**
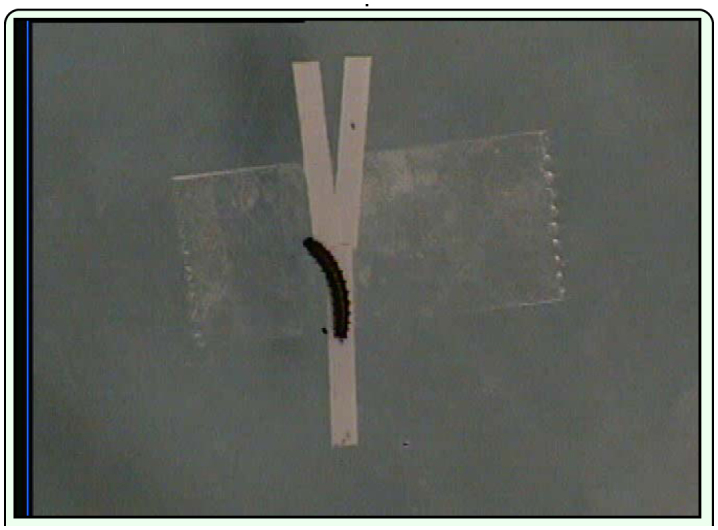
**Y** constructed from sections of a paper strip over which caterpillars of *Dione juno huascuma* had previously walked (stem and right arm). A strip not previously walked on by caterpillars (blank) forms the left arm. The response of a caterpillar allowed to choose between the arms is shown. Click image to view video. Download Video.

### Response of caterpillars to cuticular residues

**Figure 1.**
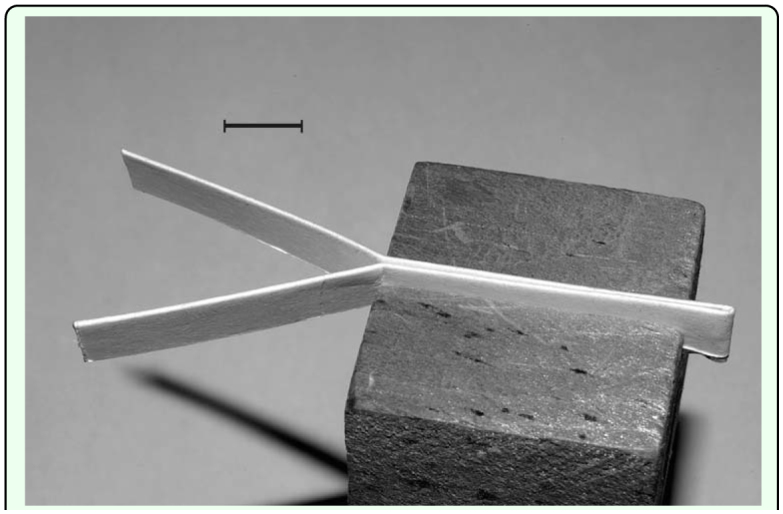
Longitudinally folded paper strip bent to form the stem and arms of a **Y.** The **Y** is inserted into a slotted wooden block. Scale Bar = cm. High quality figures are available online.

Previous studies of other species of caterpillars showed that material brushed onto the substrate when caterpillars lower and drag the ventral surfaces of the tips of the abdomens elicited trail following behavior (Costa and Pierce 1997; Costa 2006). To determine if this was also the case with *D. j. huascuma*, Y-choice tests were conducted. **Y**'s were prepared by folding a 15 cm long by 1 cm wide section of paper card longitudinally to form a double thickness strip, 0.5 cm wide. Double stick tape was used to fuse the folded halves together. Half of the folded edge of a strip was then stroked five times along the region between the anal prolegs of a fourth or fifth instar caterpillar. The strip was then formed into a Y, consisting of a stem with side by side treated and untreated pathways diverging into treated and untreated arms (Figure 1). To conduct a test, a 2^nd^ or 3^rd^ instar caterpillar was placed at the base of the stem of the **Y** and its choice of arm recorded. The maze was designed so that the caterpillar was exposed to juxtaposed pathways bearing the different treatments before reaching the choice point to assure that it compared the two treatments before selecting one over the other. A similar test was conducted comparing residue stroked onto the arms of **Y**'s from the region between the anal prolegs, as described above, and from the dorsal surface of the abdomen. **Y**'s and experimental caterpillars were used only once and treatment sides alternated to preclude a positional bias.

### Comparison of response of caterpillars to silk and cuticular residue

An experiment was conducted to compare the response of caterpillars to the arms of **Y**'s treated with either silk wound directly from the spinneret or from surface residue obtained from the region between the anal prolegs. Using a modification of a previously described procedure (Fitzgerald 1993), silk was wound onto a paper strip prepared by folding a 6 cm long by 8 mm wide section of black construction paper longitudinally and fusing the halves with double stick tape to form a double-thick strip, 4 mm wide. Black paper was used because it facilitated the visualization of the fine silk strand. The strip was mounted in the chuck of a motorized stirrer where it could be slowly rotated. A caterpillar (3^rd^ to 5^th^ instar) was held between the thumb and forefinger, and its head briefly touched to the extreme free end of the strip so that it attached a strand of silk to it. As the strip rotated in the chuck the caterpillar was moved back and forth along its exposed length so that silk issuing from the caterpillar was wound onto it. Caterpillars readily yielded continuous strands and it was possible with little effort to wind copious quantities along the exposed length of a strip. The strip was then removed from the chuck and the nonsilked, folded edge of the other half of the strip used to collect surface residue from the region between the anal prolegs as described above. The strip was then formed into a **Y** as described above, one arm of which was covered with silk and the other with cuticular residue. To conduct a test, a caterpillar was placed at the base of the stem of the **Y** and its choice of arm recorded. **Y**'s and experimental caterpillars were used only once and treatment sides alternated to preclude a positional bias.

### Response of caterpillars to silk alone

Using the procedure described above to collect silk, a Y-choice experiment was conducted to determine if silk pulled directly from the spinneret elicited a trail following response from the caterpillar. The responses of 6 second instar caterpillars to the silk bearing or blank arms of **Y**'s were recorded.

### Comparison of response of caterpillars to new and aged trails

An experiment was conducted to determine if caterpillars would respond to the arms of **Y**'s prepared by allowing 8 second instar caterpillars to walk back and forth along vertically suspended, 30 cm long strips of paper as described above, which were then aged for 24 hours. Strips were cut into 1.5 cm long sections and paired with blank strips to form the alternate arms of a Y. The stem of the **Y** was made from a strip bearing the aged pathway. Each of 10 second instar caterpillars were placed at the base of the stem of a **Y** and their choice of arm recorded. **Y**'s and experimental caterpillars were used only once. Another experiment was conducted to determine if caterpillars could distinguish between arms of **Y**'s prepared from new and aged ventral surface residue. Half of the narrow edge of a longitudinally folded strip 12 cm long by 5 cm wide as described above was prepared by stroking it along the ventral region between the anal prolegs two times. Six hours later the other half of the narrow edge of the strip was prepared in the same manner. The strip was then folded to form a **Y** and a caterpillar placed at the base of its stem and its choice of arm recorded. Arms of mazes were alternated to preclude a positional bias and were used only once.

### Comparison of response of caterpillars to trail strength

An experiment was conducted to determine if caterpillars would distinguish between pathways previously moved over by different numbers of conspecifics. A group of either 8 second instar caterpillars or a single second instar caterpillar were allowed to walk up and down separate, vertically suspended strips of paper 30 cm long × 3 mm wide for one hour. Strips were then cut into 1.5 cm long sections and used to form the arms of **Y**'s, one arm of which had been marked by the group of 8 caterpillars and the other by the single caterpillar. The stem of the maze was made from a strip marked by the group of eight caterpillars. To conduct a test, a second instar caterpillar was placed at the base of the stem of the maze and its choice of arm recorded. A similar procedure was used to prepare and test the response of caterpillars to the arms of **Y**'s previous marked by a single caterpillar and a blank, control arm.

### Species specificity of the trail marker

The response of *D. j. huascuma* caterpillars to the arms of **Y**'s previously moved over by conspecifics was compared to their response to arms previously moved over by another species of nymphalid caterpillar, *N. antiopa.* Caterpillars of the latter species were obtained from a colony maintained at UNAM Iztacala. The 1.5 cm long arms of the **Y** were cut from 30 cm long × 3 mm wide strips over which either 10 second instar *D. j. huascuma* caterpillars or 10 second instar *N. antiopa* caterpillars had been allowed to move for one-half hour as described above. The stems of the **Y**'s consisted of strips of the same length over which 10 caterpillars of both species had previously walked.

### Statistical analysis

Statistical analyses as detailed below were carried out with two-tailed χ^2^ tests with ProStat statistical software.

## Results

### Response of caterpillars to pathways of conspecifics

All of 16 caterpillars tested on **Y**'s chose the arm previously moved over by conspecifics over the blank arm. Caterpillars moved readily over the stems of the **Y**'s and typically swung their heads from arm to arm at the choice point before proceeding.

### Response of caterpillars to cuticular residues

In Y-choice experiments comparing the response of caterpillars to arms prepared from surface residue obtained from the region between the anal prolegs and untreated arms, 9 of 10 caterpillars chose the treated arm (*χ^2^* = 6.4, *P* < 0.05). When allowed to choose between arms marked with residue from the ventral prolegs and dorsal surface, 9 of 10 caterpillars selected the arms marked with the residue obtained from the region between the ventral prolegs. The results of this experiment indicate that caterpillars mark their pathways by dragging the ventral surfaces of the tips of their abdomens along the substrate and that the marker elicits trail following in conspecifics. Also, the lack of response to arms prepared from the dorsal cuticular residue indicates that the marker is not a common chemical component of the caterpillar's cuticle.

### Comparison of response of caterpillars to silk and cuticular residue

When allowed to choose between arms of **Y**'s treated with either silk pulled directly from the spinneret or residue from the region between the anal prolegs, 19 of 20 caterpillars choose the residue treated arm (*χ^2^* = 16.2, *P* < 0.001)

### Response of caterpillars to silk pulled from the spinneret

In Y-choice tests, none of six caterpillars advanced far enough along the silk treated stem to choose either the treated or blank arms. Caterpillars turned back repeatedly and advanced little from their starting position. This and the study above show that silk, per se, does not elicit a following response.

### Comparison of response of caterpillars to new and aged trails

When given a choice between the arms of a **Y** marked by caterpillars 24 hr previously and a blank arm, all of 10 caterpillars chose the previously marked arm. When allowed to choose between marked arms differing in age by 6 hrs, 12 of 13 caterpillars chose the newer arm (χ^2^ = 9.31, *P* < 0.005). The results of these two studies show that while the trail is long lived, it deteriorates with time.

### Comparison of response of caterpillars to trail strength

When given a choice between arms of **Y**'s previously marked by different numbers of conspecifics, 18 of 20 chose those marked by 8 caterpillars over those marked by 1 (χ*2* = 12.8, *P* < 0.001) and all of 15 caterpillars chose those marked by a single caterpillar over an unmarked pathway (χ^2^ = 15, *P* < 0.001). The results of this study show that individual caterpillars are capable of marking a pathway but that caterpillars follow preferentially those marked by a larger number of individuals.

### Species specificity of trail marker

When allowed to choose between the arms of **Y**'s marked by conspecifics or the caterpillars of *N. antiopa, D. j. huascuma* caterpillars chose the conspecific 14 out of 15 times (χ*2* = 11.27, *P* < 0.001). *N. antiopa* also chose the arms of mazes marked by conspecifics 12 out of 15 times (χ*2* = 5.4, *P* < 0.01). When given a choice between between a blank arm and one marked by caterpillars of the other species, *D. j. huascuma* chose the arm marked by *N. antiopa* 11 out of 15 times (χ*2* = 3.2, *P* > 0.05) and *N. antiopa* chose that marked by *D. j. huascuma* 12 out of 15 times (χ*2* = 5.4, *P* 0.025).

## Discussion

This study shows that the caterpillars of *D. j. huascuma* mark trails with a long-lived pheromone by dragging the tips of the ventral surfaces of their abdomens against the substrate. The pathways are also marked with silk and it would be reasonable to assume that the caterpillars might reference tactile or visual components of the material when moving along them. Bush (1969), for example, noted that the caterpillars of *Chlosyne lacinia* (Nymphalinae) he observed in the laboratory formed silk pathways on the inside of the container in which they were reared. The fact that the caterpillars followed each other was attributed to their ability to sense a tactile component of the silk pathway, but the possibility that a trail pheromone might serve to reinforce the tactile stimulus was also raised. Inouye and Johnson (2005) likewise stated that the caterpillars of *C. poecile* follow silk trails. In our study, the caterpillars of *D. j. huascuma* refused to move along strips bearing silk drawn directly from the spinneret, but readily followed silk-less pathways marked with the pheromone. This shows that for this species the tactile, visual, or chemical properties of silk are neither adequate nor necessary to elicit a trail-following response from the caterpillars. In a study of heliconiid caterpillars in Trinidad, Alexander (1961) suggested that silk functioned primarily to increase steadfastness on plant surfaces and as a life-line to allow caterpillars that had fallen to regain the plant. This would also appear to be the primarily function of silk in *D. j. huascuma.* It should also be noted that while Muyshondt et al. (1973) reported that the caterpillars move together in a solid column, the caterpillar is not a true processionary. When moving en masse the caterpillars are typically strung out along the trail rather than in intimate physical contact as is characteristic of processionaries.

The results of this study are consistent with similar studies of other species of the Lepidoptera showing that a trail pheromone rather than silk is the basis for trail following. Extra-silk trail pheromones have now been demonstrated for *M. americanum* (Fitzgerald and Edgerly 1982), *M. disstria* (Fitzgerald and Webster 1993), *Gloveria* sp. (Lasiocampidae) (Fitzgerald and Underwood 1998a), *M. neustrium* (Peterson 1988), *Eucheria socialis* (Pieridae) (Fitzgerald and Underwood 1998b) *Arsenura armida* (Saturniidae) (Costa et al. 2001), *Thaumetopoea* pityocampa (Thaumetopoeidae,) (Fitzgerald 2003), *Eriogaster lanestris* (Ruf et al. 2001), and *Hylesia lineata* (Saturniidae) (Fitzgerald and Pescador-Rubio 2002). In addition, the larvae of the curculionid beetle, *Phelypera distigma* have been shown to employ a trail pheromone to hold their processions together (Costa et al. 2004; Fitzgerald et al. 2004) and a trail marker has been implicated in the foraging biology of the red-headed pine sawfly, *Neodiprion leconte* (Hymenoptera: Diprionidae) (Costa and Louque 2001). In three other lepidopterans: *Hemileuca oliviae* (Saturniidae) (Capinera, 1980), *Yponomeuta cagnagellus* (Yponomeutidae) (Roessingh 1989, 1990) and *Archips* cerasivoranus (Tortricidae) (Fitzgerald 1993a) caterpillars have been shown to respond to chemical/physical properties of the silk itself with no evidence of an extra-silk pheromonal component.

While this study establishes chemical marking of a trail as the proximate mechanism for the maintenance of caterpillar aggregation in the nomadic colonies of *Dione*, too little is presently known of the behavioral ecology of the insect to enable an understanding of the functional significance of the behavior. The persistence of cohesive groups through the whole of the insect's six caterpillar stadia, terminating in group pupation (Muyshondt et al. 1973), argues strongly that the behavior offers tangible benefit to the caterpillars. In general, sociality in caterpillars has been shown to enhance fitness by facilitating foraging, defense against predators, shelter building, and thermoregulation (Fitzgerald 1993b). Only the first two of these have potential relevance to the biology of *Dione.*

Muyshondt et al. (1973) observed that aggregates of the caterpillars shook their bodies in unison and emitted a disagreeable odor when disturbed. Moreover, they noted low levels of parasitism and predation in the colonies. While the potential of group behavior for the facilitation of foraging has not been investigated in *Dione*, studies of the trail-following species *Chlosyne janais* (Nymphalidae: Melitaeini) by Denno and Benrey (2003) showed that caterpillars in larger groups grew significantly faster than those in smaller groups. A similar facilitation of feeding has been shown to occur in *C. poecile* (Inouye and Johnson 2005), but the group advantage was limited to the first caterpillar stadium; thereafter caterpillar growth rates declined with increasing group sizes. The exact mechanism leading to the facilitation of feeding was not determined for either species. The authors of both studies suggest that enhanced predator defense may also favor aggregation in these species but no studies bearing on this possibility have yet been conducted.

Beginning with the first demonstration of chemical communication in the eastern tent caterpillar (Fitzgerald 1976) the study of trail marking by caterpillars has been ongoing for over 30 years. Chemical trail marking has thus far been demonstrated in the five families of the Lepidoptera listed above and, in the present study, the Nymphalidae. Considerable progress has been made in elucidating the role of trail-based chemical communication in the collective foraging behavior of social caterpillars (see review in Costa 2006), allowing for some generalities. The pheromones are typically long-lived, species-specific, and deposited in the manner of most ants and termites when the caterpillar touches or drags the ventral surface of the tip of its abdomen along the pathway. In all species thus far studied, trail pheromones have been shown to facilitate aggregation, en masse movements of the colony from one site to another, and in the central place foragers, efficient movement between the nest and remote feeding sites. In the nomadic forest tent caterpillar, a bi-level trail marking system allows caterpillars to regroup en masse at a new bivouac after feeding (Fitzgerald and Costa 1986). Both the eastern tent caterpillar and *E. lanestris* also employ bi-level trail pheromone systems, which in the former species enable successful foragers to recruit tent mates to their food finds, much in the manner of ants (Fitzgerald 1995; Ruf et al. 2001).

Compared to the current understanding of the role of trail pheromones in the behavioral ecology of foraging by social caterpillars, little progress has been made in other areas. The specific source of a pheromone has been tentatively identified in only one species, *Hylesia lineatea* (Fitzgerald and Pescador-Rubio 2002). Moreover, chemists have shown little interest in the chemical ecology of caterpillars and in only one case has a pheromone been identified (Crump et al. 1987). In contrast, the sex pheromones of adult lepidopterans are among the most intensely studied of all the insect pheromones. This is the case because of the potential for using adult pheromones as tools for monitoring and managing pest populations. In contrast, trail pheromones have been considered to have little potential as control agents. However, it was recently demonstrated in a pilot study that when trees infested with young colonies of either the forest or eastern tent caterpillars were sprayed with a formulation of the trail pheromone mimic 5β-cholestan-3-one, the colonies disintegrated and the caterpillars perished within a few days (Fitzgerald 2008). Caterpillars are among the most destructive of all insect pests and this pilot study suggests that a better knowledge of the chemistry of their trail-based communication systems might well lead to new, ecologically sound ways to manipulate their populations.
